# Primary Coenzyme Q Deficiency in *Pdss2* Mutant Mice Causes Isolated Renal Disease

**DOI:** 10.1371/journal.pgen.1000061

**Published:** 2008-04-25

**Authors:** Min Peng, Marni J. Falk, Volker H. Haase, Rhonda King, Erzsebet Polyak, Mary Selak, Marc Yudkoff, Wayne W. Hancock, Ray Meade, Ryoichi Saiki, Adam L. Lunceford, Catherine F. Clarke, David L. Gasser

**Affiliations:** 1 Department of Genetics, University of Pennsylvania School of Medicine, Philadelphia, Pennsylvania, United States of America; 2 Department of Pediatrics, Division of Human Genetics, The Children's Hospital of Philadelphia and University of Pennsylvania School of Medicine, Philadelphia, Pennsylvania, United States of America; 3 Department of Medicine, University of Pennsylvania School of Medicine, Philadelphia, Pennsylvania, United States of America; 4 Mitochondrial Research Laboratory, Department of Pediatrics, The Children's Hospital of Philadelphia and University of Pennsylvania School of Medicine, Philadelphia, Pennsylvania, United States of America; 5 Department of Pediatrics, Division of Metabolism, Children's Hospital of Philadelphia and University of Pennsylvania School of Medicine, Philadelphia, Pennsylvania, United States of America; 6 Department of Pathology and Laboratory Medicine, Children's Hospital of Philadelphia and University of Pennsylvania School of Medicine, Philadelphia, Pennsylvania, United States of America; 7 Biomedical Imaging Core Facility, University of Pennsylvania School of Medicine, Philadelphia, Pennsylvania, United States of America; 8 Department of Chemistry and Biochemistry, University of California Los Angeles, Los Angeles, California, United States of America; Harvard Medical School, United States of America

## Abstract

Coenzyme Q (CoQ) is an essential electron carrier in the respiratory chain whose deficiency has been implicated in a wide variety of human mitochondrial disease manifestations. Its multi-step biosynthesis involves production of polyisoprenoid diphosphate in a reaction that requires the enzymes be encoded by *PDSS1* and *PDSS2*. Homozygous mutations in either of these genes, in humans, lead to severe neuromuscular disease, with nephrotic syndrome seen in *PDSS2* deficiency. We now show that a presumed autoimmune kidney disease in mice with the missense *Pdss2^kd/kd^* genotype can be attributed to a mitochondrial CoQ biosynthetic defect. Levels of CoQ_9_ and CoQ_10_ in kidney homogenates from B6.*Pdss2^kd/kd^* mutants were significantly lower than those in B6 control mice. Disease manifestations originate specifically in glomerular podocytes, as renal disease is seen in *Podocin/cre,Pdss2^loxP/loxP^* knockout mice but not in conditional knockouts targeted to renal tubular epithelium, monocytes, or hepatocytes. Liver-conditional B6.*Alb/cre,Pdss2^loxP/loxP^* knockout mice have no overt disease despite demonstration that their livers have undetectable CoQ_9_ levels, impaired respiratory capacity, and significantly altered intermediary metabolism as evidenced by transcriptional profiling and amino acid quantitation. These data suggest that disease manifestations of CoQ deficiency relate to tissue-specific respiratory capacity thresholds, with glomerular podocytes displaying the greatest sensitivity to *Pdss2* impairment.

## Author Summary

Coenzyme Q is a critical component of the mitochondrial respiratory chain, the process by which cells make energy. Coenzyme Q deficiency in humans causes a wide range of disease manifestations affecting the nervous system, muscles, and kidneys. Here, we show that the failure to make Coenzyme Q due to a *Pdss2* mutation is the cause of a lethal kidney disease in mice that was previously thought to result from an autoimmune process. Studying both a spontaneously occurring missense mutant and a series of mutants generated to have the Coenzyme Q deficiency targeted solely to liver, kidney, or macrophages, we show that the specific cell type in which the kidney disease arises is the glomerular podocyte. No other manifestations of disease are evident in these animals. However, our analysis of livers from these mice reveals that they have significant depletion of Coenzyme Q, impairment of mitochondrial respiratory chain function, and disturbance of many other basic metabolic processes. Similar microarray patterns of cellular alterations to primary mitochondrial dysfunction were seen both in these mice and in a previously reported nematode model, suggesting that a common cellular profile of primary respiratory chain function may exist across evolution.

## Introduction

Coenzyme Q (CoQ) is a benzoquinone molecule with a polyisoprenylated side chain that ranges from 6 to 10 isoprenyl units in length. It functions as an electron carrier in the mitochondrial respiratory chain, where it transports electrons from complexes I or II to complex III. The polyisoprenyl diphosphate synthases which form the isoprenyl side chain of CoQ in mice and humans are each heterotetramers of two protein subunits [1]. The genes that encode these subunits are now designated *Pdss1* and *Pdss2* in mice, and *PDSS1* and *PDSS2* in humans. Although its identity was not understood at the time, the first known mutation in the *Pdss2* gene arose spontaneously in the CBA/CaH colony of Dr. Mary Lyon and was designated kidney disease (*kd*). Homozygotes for the *kd* allele develop a lethal disease characterized by tubulointerstitial nephritis, dilated tubules, and proteinuria [2]. Mutant *kd/kd* homozygotes appear healthy for at least the first 8 weeks of life, but histological examination of the kidneys beginning at about 12 weeks of life reveals a mononuclear cell infiltrate and tubular dilatation with proteinaceous casts in cortical areas. Over time this extends to involve the entire kidney with resultant renal failure [3],[4],[5].

Renal disease pathogenesis was initially thought to be immune mediated, rather than resulting from a structural or developmental defect [3],[6]. However, we have since shown that the same renal disease, including leukocytic infiltration of macrophages and natural killer cells, develops spontaneously in *kd/kd;Rag-1^−/−^* double homozygotes lacking functional T and B lymphocytes [7]. Furthermore, mutant *kd/kd* mice are now recognized to have features of collapsing glomerulopathy (CG), a unique glomerular morphology in which hyperplastic and hypertrophic podocytes overlie collapsed capillary loops. While interstitial nephritis is often present in CG, no single definable pathogenic trigger for this disease has emerged [8]. Dysregulation of podocyte terminal differentiation in *kd/kd* mice was demonstrated by *de novo* expression of cyclin D1 (marking cell-cycle engagement) and Ki-67 (indicating podoctye cell-cycle progression), with loss of expression of differentiation markers WT-1 and synaptopodin [9]. These results suggested that there may indeed be an intrinsic structural defect in the podocytes of *kd/kd* mice, with the inflammatory reaction playing only a secondary role.

A positional cloning approach demonstrated that the *kd* allele is a missense mutation in a prenyltransferase-like mitochondrial protein [10], now designated *Pdss2.* This enzyme forms a heterotetramer with another enzyme encoded by *Pdss1* to generate nonaprenyl diphosphate in mice or decaprenyl diphosphate in humans. The polyisoprenoid diphosphate precursor is added to 4-hydroxybenzoic acid, forming polyisoprenyl-hydroxybenzoic acid, an early intermediate in CoQ biosynthesis [1]. Compound heterozygous *PDSS2* mutations were recently identified in a child with severe Leigh syndrome and nephrotic syndrome [11]. Primary CoQ deficiency in humans is a rare condition that typically presents with neurologic or myopathic disease that is often ameliorated with CoQ supplementation [12]. It has now been associated with mutations in three of the nine nuclear genes encoding component enzymes of the CoQ biosynthesis pathway including *COQ2*, *PDSS1*, and *PDSS2* [11],[13],[14],[15] as well as with electron-transferring-flavoprotein dehydrogenase (*ETFDH*) mutations affecting the transfer of electrons to CoQ [16]. Nephrotic syndrome has been seen in individuals with mutations in *PDSS2* [11], as well as *COQ2* [13],[14],[17]. Mechanisms underlying tissue-specific disease manifestations remain unclear.

We report here results of extensive phenotypic characterizations of both *kd/kd* (B6.*Pdss2^kd/kd^*) missense and tissue-specific conditional *Pdss2* knockout mice, including those affecting renal glomerular podocytes (B6.*Podocin/cre,Pdss2^loxP/loxP^*), renal tubular epithelium and hepatocytes (B6.*PEPCK/cre,Pdss2^loxP/loxP^*), monocytes (B6.*LysM/cre,Pdss2^loxP/loxP^*), and hepatocytes (B6.*Alb/cre,Pdss2^loxP/loxP^*). This work demonstrates that renal disease in *Pdss2^kd/kd^* mice results from podocyte-specific *Pdss2* dysfunction. In addition, while biochemical evidence of significant CoQ deficiency, respiratory chain dysfunction in isolated mitochondria, and altered amino acid profiles is present in livers of both B6.*Pdss2^kd/kd^* missense and B6.*Alb/cre,Pdss2^loxP/loxP^* mutant mice, no overt manifestations of liver or other extra-renal disease were observed in any of the mutants through eight months of life.

## Results

### *Pdss2^kd/kd^* Phenotype Is Recapitulated in B6.*Podocin/cre,Pdss2^loxP/loxP^* Mice

**Figure 1:**
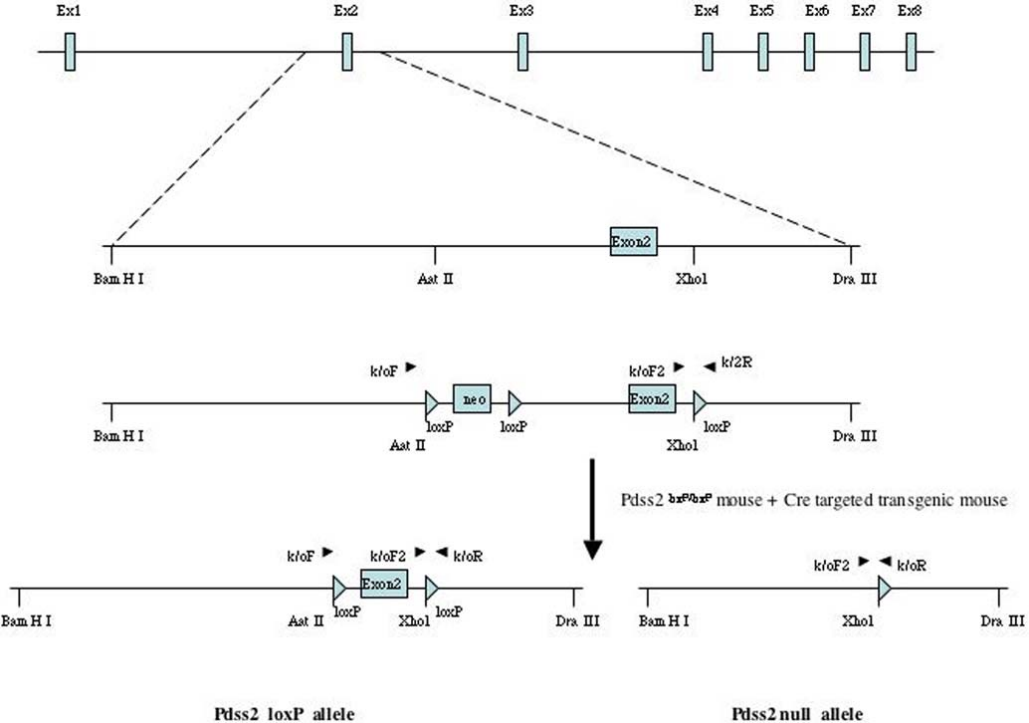
Generation of a *Pdss2* Conditional Null Allele. Generation of a *Pdss2* conditional null allele, showing a map of the *Pdss2* genomic locus and the targeting vector with exons represented by open boxes. The relative position of PCR primers (small arrowheads), loxP (large arrowheads), as well as cassettes encoding neomycin phosphotransferase (neo) are shown. Primers koF, koF2 and koR were used in PCR genotype analysis. Cre-mediated deletion results in either the *Pdss2* null allele (deletion of exon 3 ) or the *Pdss2 ^loxP/loxP^* allele (exon 2 flanked by loxP sites). Abbreviations: Ba, BamH I; Aa. Aat II; Xh, Xho I; Dr, Dra III.

**Figure 2:**
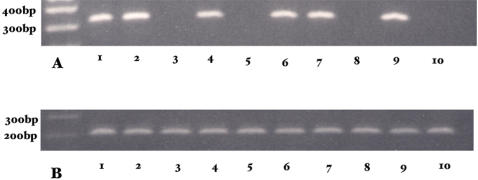
*Pdss2* Conditional Knockout Confirmation. PCR products after laser capture microdissection, using primers for exon 2 (A) or exon 4 (B). Lanes: 1, *Pdss2^loxP/loxP^* glomerulus; 2, *Pdss2^loxP/loxP^* tubules; 3, B6.*Podocin/cre,Pdss2^loxP/loxP^* mouse 1, glomerulus; 4, B6.*Podocin/cre,Pdss2^loxP/loxP^* mouse 1 tubules; 5, B6.*Podocin/cre,Pdss2^loxP/loxP^* mouse 2, glomerulus; 6, B6.*Podocin/cre,Pdss2^loxP/loxP^* mouse 2 tubules; 7, B6.*PEPCK/cre*,*Pdss2^loxP/loxP^* mouse 1 glomerulus; 8, B6.*PEPCK/cre,Pdss2^loxP/loxP^* mouse 1, tubules; 9, B6.*PEPCK/cre*,*Pdss2^loxP/loxP^* mouse 2 glomerulus; 10, B6.*PEPCK/cre,Pdss2^loxP/loxP^* mouse 2, tubules.

The *Pdss2^loxP^* construct was prepared as shown in Figure 1. The total knockout (B6.*Zp3/Cre*,*Pdss2^loxP/loxP^*) was embryonically lethal , with no homozygous embryos surviving beyond 10.5 days of gestation (data not shown). This is in agreement with the findings of Levavasseur *et al*. [18] and Nakai *et al*. [19], who reported that mouse embryos deficient in CoQ synthesis as a result of *clk-1/coq7* (demethoxyubiquinone hydroxylase) mutations also arrest development at mid-gestation. Tissue-specific conditional knockouts were therefore generated by crossing B6.*Pdss2^loxP/loxP^* mice with several *Cre*-expressing strains. As shown in Figure 2, the *Pdss2* gene was successfully targeted in the glomeruli but not the collecting tubules of B6.*Podocin/cre*,*Pdss2^loxP/loxP^* mice, whereas the collecting tubules and hepatocytes but not the glomeruli were targeted in B6.*PEPCK/cre*,*Pdss2^loxP/loxP^* mice.

**Figure 3:**
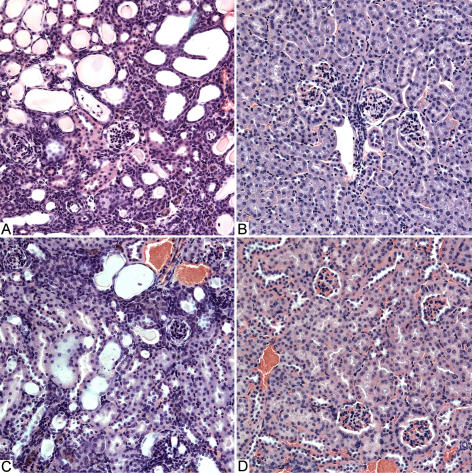
Histologic Features of Renal Disease in *Pdss2* Mutant and Control Mice. Histologic features of renal disease in mutant and control mice (H & E-stained sections, original magnifications all 200x). *A*, B6.*Podocin/cre,Pdss2^loxP/loxP^* mouse (290 days old; 64 mg albumin; histologic score, 4+). *B,* B6.*PEPCK/cre*, *Pdss2^loxP/loxP^* (191 days old; 0.04 mg albumin; histologic score 0). *C*, B6.*Pdss2^kd/kd^* (146 days old; 15 mg albumin; histologic score, 3+). *D*, B6. *Pdss2^loxP/loxP^* mouse, (191 days old; 0.12 mg albumin; histologic score, 0). Note the prominent tubular dilatation and interstitial infiltrates in panels A and C, but absent in panels B and D.

**Figure 4:**
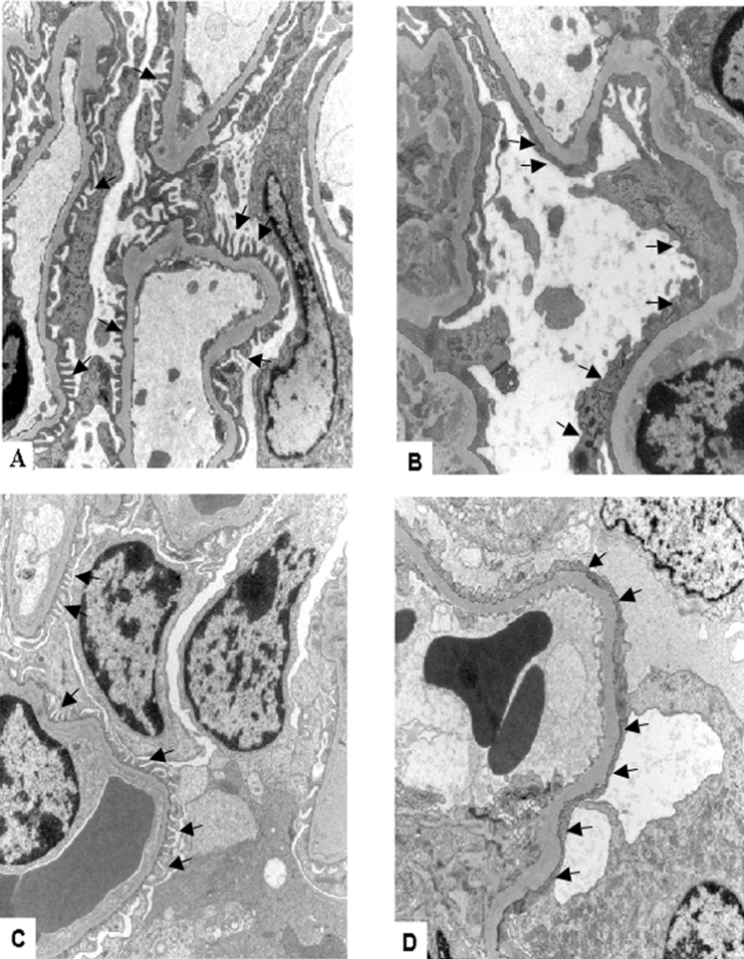
Glomerular Electron Micrographs From *Pdss2* Mutant and Control Mice. Electron micrographs from mutant and control mouse kidney glomeruli; original magnifications all 10,000x; scale bar = 2 microns. *A*, B6, 362 days old; arrows show podocyte foot processes. *B,* B6.*Pdss2^kd/kd^*, 267 days old; arrows show regions of foot process effacement. *C*, B6.*Pdss2^loxP/loxP^* mouse, 248 days old; arrows show foot processes. *D*, B6.*Podocin/cre,Pdss2^loxP/loxP^* 290 days old; arrows show regions of foot process effacement.

B6.*Podocin/cre,Pdss2^loxP/loxP^* but not B6.*PEPCK/cre*,*Pdss2^loxP/loxP^* mice had the same kidney disease phenotype as B6.*Pdss2^kd/kd^* missense mice, as judged by albuminuria and histological evidence of nephritis. Sections of kidneys from B6.*Podocin/cre*,*Pdss2^loxP/loxP^* mice, B6.*PEPCK/cre*,*Pdss2^loxP/loxP^* mice, and controls are shown in Figure 3. The phenotype of B6.*Podocin/cre*,*Pdss2^loxP/loxP^* is histologically indistinguishable from that of a B6.*Pdss2^kd/kd^* missense mutant, with both showing greatly dilated tubules and extensive interstitial infiltration. In contrast, neither feature is seen in the B6.*PEPCK/cre*,*Pdss2^loxP/loxP^* or the B6.*Pdss2^loxP/loxP^* control. Renal tissue from conditional knockouts was also examined by electron microscopy, with results shown in Figure 4. No significant ultrastructural differences were observed between B6 and B6.*Pdss2^loxP/loxP^* control kidneys (Figures 4A and 4C), but the abnormalities present in the B6.*Podocin/cre,Pdss2^loxP/loxP^* knockout (Figure 4D) were the most extensive of any *Pdss2* mutant studied [10]. Measurements of 24-hour urine albumin and semi-quantitative histological scores from *Pdss2* missense, all conditional knockout mutants, as well as controls, are shown in Table 1. Only the B6.*Podocin/cre,Pdss2^loxP/loxP^* knockouts had a phenotype that resembled that of the B6.*Pdss2^kd/kd^* mice, as measured by either albuminuria or histologically-scored nephritis.

**Table 1:** Phenotypes of mice with conditional knockout and control genotypes*

Genotype	Urine albumin (mg/24 hrs)	Nephritis
B6.*Podocin/cre,Pdss2^loxP/loxP^*	32.89 +/− 7.64 (28)**	1.80 +/− 0.29 (26)
B6.*PEPCK/cre*, *Pdss2^loxP/loxP^*	0.18 +/− 0.05 (18)	0.05 +/− 0.05 (20)
B6.*LysM/cre*, *Pdss2^loxP/loxP^*	0.12 +/− 0.01 (6)	0.33 +/− 0.08 (6)
B6.*Alb/cre,Pdss2^loxP/loxP^*	0.13 +/− 0.03 (6)	0.17 +/− 0.15 (6)
B6.*Pdss2^kd/kd^*	21.17 +/− 5.41 (6)	2.11 +/− 0.40 (9)
B6. *Pdss2^loxP/loxP^*	0.24 +/− 0.07 (7)	0.73 +/− 0.22 (11)
B6	0.12 +/− 0.01 (8)	0.58 +/− 0.18 (12)

*: All mice at least 120 days old.

**: Mean +/− SEM; N is shown in parentheses.

Another phenotypic abnormality seen in B6.*Pdss2^kd/kd^* homozygotes is elevated serum cholesterol and triglycerides [20]. In an effort to determine whether this alteration may in part result from the underlying biochemical defect in CoQ biosynthesis rather than wholly as a consequence of nephrotic syndrome, we measured cholesterol and triglyceride levels in B6.*Podocin/cre*,*Pdss2^loxP/loxP^* and B6.*Alb/cre,Pdss2^loxP/loxP^* mice. B6.*Podocin/cre,Pdss2^loxP/loxP^* mice developed renal disease associated with significantly elevated plasma cholesterol levels (Table 2), suggesting hypercholesterolemia is largely a consequence of nephrotic syndrome. However, significantly elevated plasma cholesterol was also seen in B6.*Alb/cre,Pdss2^loxP/loxP^* (94 +/− 7.1 mg/dl) when compared with B6.*Pdss2^loxP/loxP^* controls (61 +/− 5.5 mg/dl), suggesting that a *Pdss2* defect in the hepatocytes does contribute to hypercholesterolemia.

**Table 2:** Plasma cholesterol levels in mice with conditional knockout and control genotypes

*Genotype*	Plasma cholesterol (mg/dl)
B6.*Podocin/cre,Pdss2^loxP/loxP^*	144 +/− 21.3 (7)
B6.*Alb/cre,Pdss2^loxP/loxP^*	94 +/− 7.1 (7)
B6. *Pdss2^loxP/loxP^*	61 +/− 5.5 (7)

All mice were at least 120 days old; data express mean +/− SEM; N is shownin parentheses. For B6.*Podocin/cre,Pdss2^loxP/loxP^ vs.* B6. *Pdss2^loxP/loxP^*, t = 3.50 and p = 0.004; for B6.*Podocin/cre,Pdss2^loxP/loxP^ vs.* B6.*Alb/crePdss2^loxP/loxP^*, t = 2.09 and p = 0.058; for B6.*Alb/cre,Pdss2^loxP/loxP^ vs.* B6. *Pdss2^loxP/loxP^*, t = 3.34 and p = 0.006.

### *Pdss2* Mutations Diminish CoQ Tissue Levels

**Figure 5:**
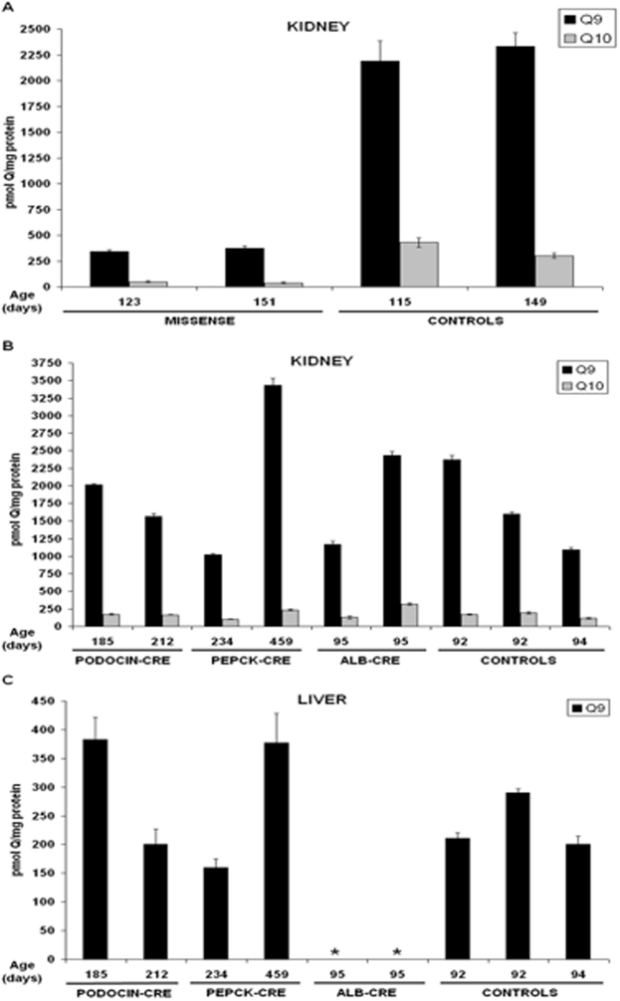
CoQ Measurements in *Pdss2* Missense, Conditional Knockout, and Strain-Matched Control Mice. CoQ measurements in *Pdss2^kd/kd^*; *Podocin/cre,Pdss2^loxPloxP^*; *PEPCK/cre,Pdss2^loxPloxP^*; *Alb/cre,Pdss2^loxP/loxP^*; and strain-matched control mice (B6 and B6.*Pdss2^loxPloxP^*). *A*, Kidney levels of CoQ_9_ and CoQ_10_ are significantly lower in B6.*Pdss2^kd/kd^* (missense) mice than in B6 control mice. *B*, Kidney levels of CoQ_9_ and CoQ_10_ are similar in B6.*Podocin/cre,Pdss2^loxPloxP^*; *B6.PEPCK/cre,Pdss2^loxPloxP^*; B6.*Alb/cre,Pdss2^loxP/loxP^*; and control mice. The 92-day-old controls are B6.*Pdss2^loxP/+^*, and the 94-day-old control is B6.*Pdss2^loxP/loxP^*. *C*, Liver levels of CoQ_9_ in B6.*Alb/cre,Pdss2^loxP/loxP^* mice were below 30 picomoles/mg protein (*) while these same levels in B6.*Podocin/cre,Pdss2^loxPloxP^* and B6.*PEPCK/cre,Pdss2^loxPloxP^* mice were similar to those in control mice. The strains of the control mice in are the same as those in panel *B*.

CoQ content was determined in lipid extracts of tissue homogenates from livers and kidneys dissected from mutant and control mice. As shown in Figure 5 (Panel A), there was a significant reduction in CoQ_9_ and CoQ_10_ levels in the kidneys of B6.*Pdss2^kd/kd^* mice compared to age-matched B6 controls. There were no significant differences in kidney CoQ_9_ content between the B6, B6.*Pdss2^loxP/+^*, or B6.*Pdss2^loxP/loxP^* control mice (Panels A and B). Neither the B6.*Podocin/cre*,*Pdss2^loxP/loxP^* nor the B6.*PEPCK/cre*,*Pdss2^loxP/loxP^* mice had a significant reduction in the CoQ_9_ levels of total liver or kidney homogenates (Panels B and C), which is consistent with the fact that only a small subset of cells were affected by these targeted disruptions. However, the B6.*Alb/cre,Pdss2^loxP/loxP^* mice had less than 30 pmol CoQ_9_ per mg liver protein, which would be expected if most hepatic cells were affected by the albumin promoter-driven *Cre* expression.

### *Pdss2* Mutations Do Not Consistently Alter *Pdss1* Expression in Liver or Whole Kidney

Relative quantitation expression studies in isolated liver tissue demonstrated significant *Pdss2* knock-down in each of three B6.*Alb/cre,Pdss2^loxP/loxP^* mutants (mean 97.2% decrease; range 97.0% to 97.3% decrease), but no consistent change in nine B6.*Pdss2^kd/kd^* missense mice (mean 6% increase; range 40% decrease to 250% increase) in comparison with appropriate strain- and age-matched pooled controls. *Pdss1* expression showed inconsistent alterations both among six B6.*Alb/cre,Pdss2^loxP/loxP^* mutants (mean 21% increase; range 50% decrease to 180% increase) as well as nine B6.*Pdss2^kd/kd^* missense mutants (mean 4% decrease; range 22% decrease to 57% increase) in comparison with pooled *Pdss2^loxP/^* ^loxP^ or B6 respective controls. Finally, relative quantitation demonstrated a decrease in expression of both *Pdss1* and *Pdss2* transcripts in RNA isolated from intact single kidneys of three B6.*Pdss2^kd/kd^* missense mutants with renal disease [(*Pdss1* mean 71% decrease; range 65% to 80% decrease) and (*Pdss2* mean 35% decrease; range 26% to 44% decrease)], but no consistent alteration of either transcript in RNA isolated from intact single kidneys of three B6.*Podocin/cre,Pdss2^loxP/loxP^* mutants with renal disease [(*Pdss1* mean 31% decrease; range 75% decrease to 55% increase) and (*Pdss2* mean 17% increase; range 4% decrease to 57% increase)].

### *Pdss2* Mutations Impair Mitochondrial Respiratory Chain Function

**Figure 6:**
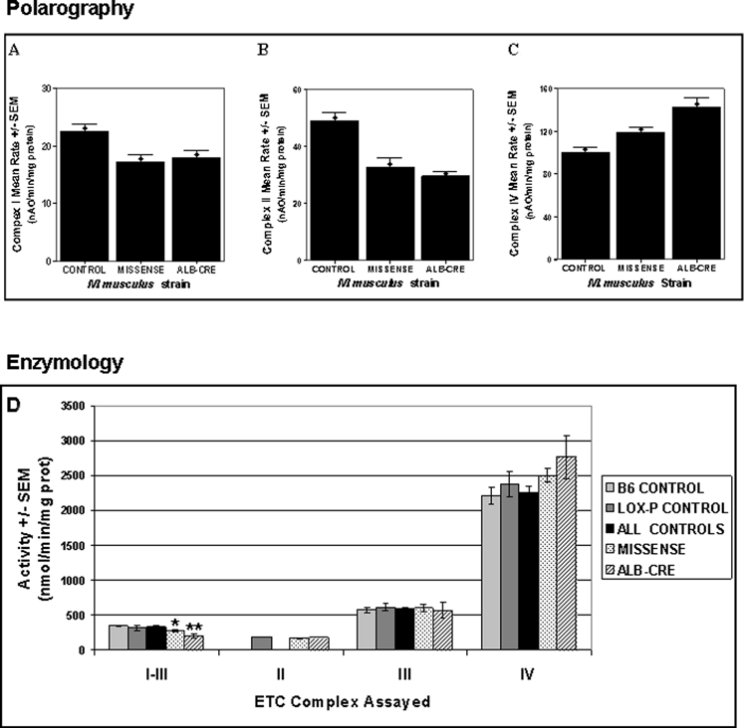
CoQ Deficiency Causes Mitochondrial Dysfunction in *Pdss2* Mutant Mice. Impact of coenzyme Q deficiency on mitochondrial function in B6.*Pdss2^kd/kd^* and B6.*Alb/cre,Pdss2^loxP/loxP^* mice compared to concurrent age- and strain-matched controls (B6 and B6.*Pdss2^loxP/loxP^*, respectively). Panels A, B, and C demonstrate polarographic results of freshly isolated liver mitochondria with substrates which specifically interrogate complex I-dependent (malate+glutamate), complex II-dependent (succinate), and complex IV-dependent (TMPD+ascorbate) OXPHOS capacity. B6.*Pdss2^kd/kd^* missense mutants have *impaired* complex I- and complex II-dependent OXPHOS capacity by 23% and 33%, respectively, with a 19% *increase* in complex IV-dependent OXPHOS capacity. B6.*Alb/cre,Pdss2^loxP/loxP^* mutants have *impaired* complex I- and complex II-dependent OXPHOS capacity by 20% and 40% respectively, with a 43% *increase* in complex IV-dependent OXPHOS capacity. Values represent state 3 (near maximal) oxygen consumption mean rate and standard error. Panel D summarizes electron transport chain activity assays for complexes I-III, II, III, and IV performed on remaining aliquots of liver mitochondria frozen following completion of polarography. Similar to polarographic results, I-III enzyme activity is significantly decreased by 19% in B6.*Pdss2^kd/kd^* missense mutants and by 41% in B6.*Alb/cre,Pdss2^loxP/loxP^* mutants when compared to pooled controls. Complex IV enzyme activity demonstrates a trend toward increase by 11% in B6.*Pdss2^kd/kd^* missense mutants and by 23% in B6.*Alb/cre,Pdss2^loxP/loxP^* mutants when compared to pooled controls, but does not reach the level of statistical significance, perhaps because of small sample size (n = 2 for remaining mitochondria from B6.*Alb/cre,Pdss2^loxP/loxP^* mutants). As expected, no change is seen in complex II or III activity for either mutant. Values represent mean enzyme activity and standard error. B6 and B6.*Pdss2^loxP/loxP^* ETC activity values were pooled to increase control sample size following demonstration of no significant differences between these strains. Statistical analyses comparing mutants and controls were performed by one-way ANOVA, where * indicates p<0.05 and ** indicates p<0.01.

B6.*Alb/cre,Pdss2^loxP/loxP^* mice had no evidence of disease through 8 months of life, but isolated liver mitochondria respiratory chain capacity in 6 to 8 month old animals was impaired to a similar extent as seen in B6.*Pdss2^kd/kd^* missense mice. Specifically, polarography of freshly isolated liver mitochondria showed significantly decreased complex I- and complex II-dependent integrated respiratory chain capacity in both B6.*Pdss2^kd/kd^* and B6.*Alb/cre,Pdss2^loxP/loxP^* mutants compared with controls (Figure 6, panels A and B). Significantly *increased* complex IV-dependent respiratory capacity was also observed in both the B6.*Pdss2^kd/kd^* and B6.*Alb/cre,Pdss2^loxP/loxP^* mutants (Figure 6, panel C). This suggests that the degree of CoQ deficiency in liver is sufficient to cause secondary upregulation of respiratory chain components (complex IV) distal to the genetic deficiency (CoQ). These functional alterations are supported by spectrophotometric electron transport chain enzyme activity analyses performed on frozen liver mitochondria from these same animals (Figure 6, Panel D). Frozen mitochondria isolated from single whole kidney homogenates were also studied from five B6 and three B6.*Pdss2^loxP/loxP^* controls, as well as three animals each of genotypes B6.*Pdss2^kd/kd^*, B6.*Podocin/cre,Pdss2^loxP/loxP^*, B6.*PEPCK/cre*, *Pdss2^loxP/loxP^*, and B6.*Alb/cre,Pdss2^loxP/loxP^*. No significant differences were detected in activities of enzyme complex I-III, II-III, III, or IV normalized to citrate synthase activity for any of the mutants in comparison with controls, although a trend toward increase was observed for complex IV enzyme activity in B6.*Pdss2^kd/kd^* kidney mitochondria (data not shown).

### Metabolic Effects of *Pdss2* Liver Conditional Knockout

**Figure 7:**
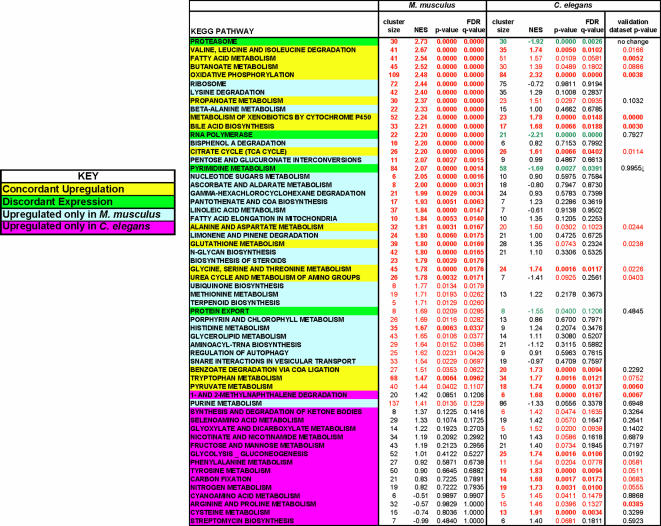
Metabolic Pathway Alterations Are Seen by Expression Profiling in B6.*Alb/cre,Pdss2^loxP/loxP^* Mouse Liver. Global genome expression profiling in B6.*Alb/cre,Pdss2^loxP/loxP^* mouse liver identifies concordant transcriptional alterations interpretable at the level of multiple metabolic pathways, which suggest significantly altered intermediary metabolism occurs despite an apparent absence of symptomatic disease. Extensive evolutionary concordance in upregulation of key biochemical pathways is seen in primary mitochondrial dysfunction, both in this mammalian *Pdss2* liver-conditional knockout model of coenzyme Q deficiency and in a previously reported *C. elegans gas-1(fc21)* missense mutant model of primary complex I dysfunction [21]. Biochemical pathways as curated from the KEGG online database (http://genome.jp.kegg) are indicated with the # of genes in each pathway (cluster size), normalized enrichment score (NES), statistical significance of altered pathway expression between mutant and wildtype controls (*p*-value), and false positive percentage in the form of a false discovery rate (FDR *q*-value) according to GSEA. Pathways are ranked by descending NES in the *Pdss2* mutant (left data column). Comparison to previously reported complex I *gas-1*(*fc21*) missense *C. elegans* mutant dataset (middle data column) and a “validation” *C. elegans* dataset of 8 different complex I, II, and III missense and RNAi-interference generated mutants (right data column) is indicated by differential highlights [21]. Font color denotes a pathway as relatively upregulated (red), downregulated (green), or unchanged (black).

A significantly altered metabolic phenotype at the level of concordant biochemical pathway expression changes was clearly present in B6.*Alb/cre,Pdss2^loxP/loxP^* mutants by 6 months of life, as summarized in Figure 7 (left column). Specific metabolic consequences of *Pdss2*-based CoQ deficiency include significant upregulation at a nominal p-value<0.05 in 43 of 95 essential KEGG biochemical pathways analyzed by Gene Set Enrichment Analysis including oxidative phosphorylation itself, the tricarboxylic acid (TCA) cycle, and multiple metabolic pathways that provide substrates necessary for energy production including fatty acid metabolism and 8 different amino acid metabolic pathways. Many of these same pathways were similarly upregulated upon expression profiling of *C. elegans* mitochondrial mutants in nuclear-encoded respiratory chain subunits of complexes I, II, and III (Figure 7, middle and right columns) [21].

Unique to the CoQ biosynthetic defect, B6.*Alb/cre,Pdss2^loxP/loxP^* mutants have upregulation of CoQ biosynthesis, likely in compensation for their underlying CoQ deficiency. Similarly, the substrates immediately prior to the *Pdss2*-encoded prenyldiphosphate synthase enzymatic block (such as farnesyl pyrophosphate) appear to get funneled toward alternative biochemical pathways, as above. Interestingly, regulation of autophagy is uniquely upregulated in the B6.*Alb/cre,Pdss2^loxP/loxP^* mutants, supporting previously reported findings of mitophagy occurring in these animals as characterized by endoplasmic reticulum engulfing liver mitochondria [10]. Consistent with CoQ function in both prooxidant and antioxidant roles, global expression analysis demonstrates the upregulation of multiple cellular defense pathways including the metabolism of P450 and glutathione, ascorbate metabolism, as well as the degradation of bisphenol A, limonene, pinene, and gamma-hexachlorocyclohexane. These mutants also appear to have alterations in basic DNA metabolism, as evidenced by upregulation of the metabolism of purines, pyrimidines, nucleotide sugars, ribosomes, aminoacyl-tRNAs, and RNA polymerase. Finally, four KEGG pathways appear on initial inspection to be discordantly expressed between mouse *Pdss2* mutants and *C. elegans* complex I subunit mutants (Figure 7, left and middle columns, green highlight); however, none of these pathways upregulated in B6.*Alb/cre,Pdss2^loxP/loxP^* were validated as having altered expression in the independent *C. elegans* dataset comprised of eight different complex I, II, and III respiratory chain subunit mutants (Figure 7, right column).

**Figure 8:**
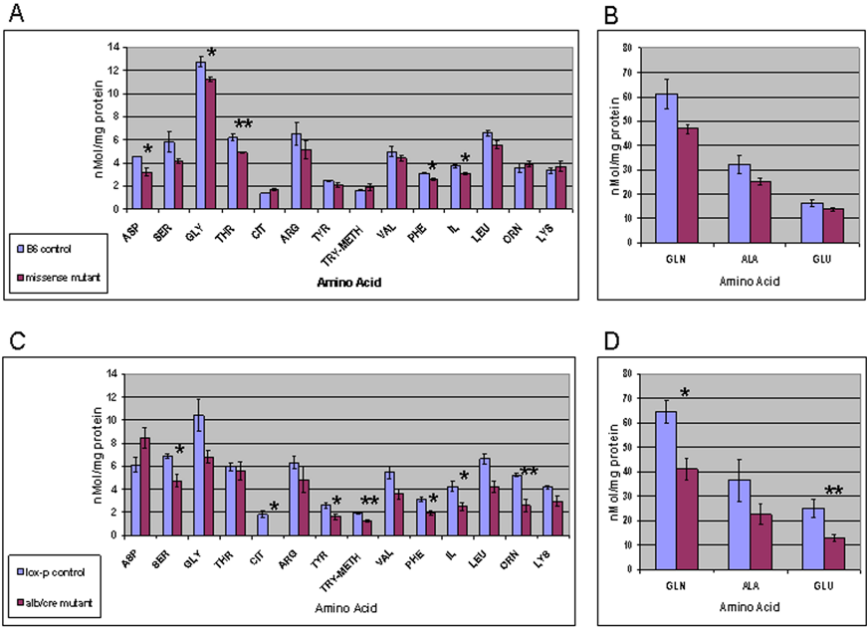
*Pdss2* Mutants Have Altered Amino Acid Profiles in Liver. *Pdss2* mutants have altered amino acid profiles in liver. Quantitative liver amino acid analysis detects significant differences in B6.*Pdss2^kd/kd^* missense mutants compared with B6 controls (Panels A and B), as well as in B6.*Alb/cre,Pdss2^loxP/loxP^* mutants compared with B6.*Pdss2^loxP/loxP^* controls (Panels C and D). To better demonstrate differences in all amino acids, results for three amino acids in highest abundance are shown separately (Panels B and D) with a greater scale compared to that used for the remainder of the amino acids present in relatively lower abundance (Panels A and C). Values represent mean +/− SEM. Statistical analyses comparing mutants and controls were performed by Student's t-test, where * indicates p<0.05 and ** indicates p<0.01.

Altered amino acid metabolism is further supported by significant concentration differences among multiple amino acids quantified in livers of B6.*Alb/cre,Pdss2^loxP/loxP^* mutants (Figure 8, Panels C and D). Hepatic glutamate was lowered to less than half the control value (p<0.01; Figure 8, Panel D). Glutamate depletion could account for decreased levels of glutamine and alanine (Figure 8, Panel D) that derive from glutamate via glutamine synthetase and alanine aminotransferase, respectively. Similarly, a loss of glutamate could lower hepatic concentrations of phenyalanine, methionine, leucine, isoleucine and valine (Figure 8, Panel C), each of which forms from glutamate by a specific transaminase. The total absence of citrulline (Figure 8, Panel C) suggests ureagenesis is compromised in *Pdss2* mutant mice. Similarly, the sole amino acid to be significantly increased was the urea cycle precursor, aspartate (Figure 8, Panel C), with a sharp reduction in the glutamate∶aspartate ratio (1∶4 vs 4∶1) of mutant vs control liver.

### *Pdss2* Missense Mutants Have No Clinical Manifestations of Extra-Renal Disease

Histological sections of the brain, retina, liver, and skeletal muscle of B6.*Pdss2^kd/k^* ^d^ mutants demonstrated no obvious structural abnormalities. Skeletal muscle sections stained with Gomori/trichrome and antibodies to muscle enzymes (ATPase, NADH, SDH) showed no evidence of subsarcolemmal mitochondrial aggregates or other abnormalities. In addition, no differences were observed in bone mineral density or concentration, lean body mass, complete blood count, or liver transaminase (ALT) (data not shown). Several significant differences were noted in the liver amino acid levels of these animals (Figure 8, Panels A and B), but these changes were not as intense or widespread as observed in livers of B6.*Alb/cre,Pdss2^loxP/loxP^* mutants (Figure 8 Panels C and D). The fact that most of these changes affected essential amino acids – threonine, phenylalanine and isoleucine, for example – could reflect albuminuria with concomitant nitrogen wasting and relative protein deficiency in these animals.

## Discussion

The occurrence of a kidney disease phenotype in B6.*Podocin/cre,Pdss2^loxP/loxP^* but not B6.*PEPCK/cre*,*Pdss2^loxP/loxP^* mice resolves a long-standing question regarding the cellular localization of the primary defect in B6.*Pdss2^kd/kd^* mice [3],[20]. Table 1 shows that a primary defect in podocytes recapitulates the *kd/kd* disease phenotype and is responsible for the development of nephrotic syndrome, which was not the case for mice expressing the defect primarily in renal tubular epithelium and hepatocytes (B6.*PEPCK/cre*,*Pdss2^loxP/loxP^*). Hence, interstitial nephritis occurs as a consequence of a *Pdss2* defect in glomerular podocytes. Knocking out the *Pdss2* gene in podocytes resulted in a more severe phenotype than that observed in mice homozygous for the *Pdss2^kd^* mutation (Figure 4), as would be expected if the product of the missense allele has at least some residual activity. Detection of CoQ_9_ and CoQ_10_ in the kidneys of B6.*Pdss2^kd/kd^* mice, while almost ten-fold lower than age-matched B6 controls, suggests that the V117M amino acid substitution mutation in the *Pdss2^kd^* gene product retains partial activity (Figure 5).

That tubular dilatation and interstitial nephritis are downstream consequences of a CoQ deficiency in podocytes is illustrated by Figure 3. Our results suggest that CoQ deficiency negatively impacts mitochondrial respiratory chain function, although loss of CoQ antioxidant function may also be involved in pathogenesis, which leads to podocyte death with important consequences for kidney function. The slit diaphragm, which is dependent on podocyte foot processes, is essentially destroyed, causing leakage of various proteins normally maintained within the circulation, such as serum albumin. Some of these molecules have deleterious effects on the collecting tubules, which leads to tubular dilatation and dysfunction, release of inflammatory mediators, and development of interstitial nephritis.

The results in Table 2 suggest that the elevation in serum cholesterol that is characteristic of *Pdss2^kd/kd^* homozygotes [20] is not entirely attributable to kidney disease, but likely results from shunting of farnesyl diphosphate (one of the precursors in the CoQ biosynthetic pathway immediately proximal to the *Pdss2* enzymatic block) to alternative pathways, primarily involving cholesterol biosynthesis. This is supported by transcriptional profiling of B6.*Alb/cre,Pdss2^loxP/loxP^* liver which demonstrated upregulation of two pathways for which cholesterol is the starting substrate, namely bile acid biosynthesis and steroid biosynthesis (Figure 7).

In light of the severe, multi-system phenotype of the child with primary CoQ deficiency due to *PDSS2* mutations [11], we undertook an extensive phenotypic evaluation of B6.*Pdss2^kd/k^* ^d^ spontaneous missense homozygous mutants. All studies were performed in animals more than 120 days old, the time by which renal dysfunction is present. No overt non-renal disease manifestations could be detected, although significant deficiencies were observed in their respiratory function and activity of isolated liver mitochondria (Figure 6), as well as quantitative amino acid profiles in liver (Figure 8). It remains possible that additional manifestations of CoQ deficiency would develop with time but do not because of the high mortality of their renal disease. This possibility is being further evaluated by prolonged observation of tissue-specific conditional knockouts without renal disease.

Interestingly, B6.*Alb/cre,Pdss2^loxP/loxP^* liver-conditional knockout mutants manifest no overt symptoms or signs of liver disease through at least eight months of life. Nonetheless, focused evaluations of the livers of these animals demonstrated substantial (97%) knockdown of *Pdss2* expression; pronounced CoQ_9_ deficiency (Figure 5) and significant impairment of complex I- and complex II-dependent integrated respiratory capacity as well as complex I-III and II-III enzyme activities with compensatory increase in complex IV-dependent respiratory capacity in isolated mitochondria (Figure 6). The relatively robust respiratory electron transport chain activities are quite remarkable, given that CoQ_9_ was not detected over background in these liver lipid extracts. The low levels of CoQ_9_ present in these animals must support significant activities of complexes I, II, and III. Very small amounts of CoQ support significant respiratory chain activity in other models (yeast, nematode, isolated mitochondria). Liu et al. [22] showed that hepatocytes in *clk-1/+* heterozygous mice lose heterozygosity (become *clk-1* −*/*−), and these cells undergo clonal expansion within the liver. These findings suggest that liver cells perform quite adequately with low CoQ content.

A significantly altered cellular metabolism phenotype in liver of B6.*Alb/cre,Pdss2^loxP/loxP^* mice was evidenced by concordant upregulation of 43 biochemical pathways (Figure 7) with significant deficiency of 9 amino acids (Figure 8). Taken together, these alterations confirm that *Pdss2* dysfunction is a nuclear gene-based mitochondrial respiratory chain defect to which cellular adaptation is occurring. Indeed, 15 key biochemical pathways were concordantly upregulated on transcriptional profiling in this murine model of primary mitochondrial dysfunction and in previously reported *C. elegans* models of primary respiratory chain dysfunction due to mutations in nuclear gene-encoded subunits of complexes I, II, and III (Figure 7). These similarities spanning evolution provides support that a transcriptional “signature” of mutations in the respiratory chain appears to be stimulation of the constituent components of oxidative phosphorylation, the enzymes of the TCA cycle, and many pathways (e.g., glycolysis, amino acid metabolism, fatty acid metabolism) that furnish substrate to it, as well as stimulation of key cellular defense pathways such as glutathione and P450 metabolism [21].

Global amino acid alterations in *Pdss2* mutant liver further suggest that significant impairment may be occurring in flux through key biochemical pathways, including glycolysis, the pyruvate dehydrogenase reaction, the TCA cyle, and ureagenesis. Changes in the hepatic amino acid profile were not qualitatively dissimilar in B6.*Alb/cre,Pdss2^loxP/loxP^* mutants compared with those of the B6.*Pdss2^kd/k^* ^d^ missense mutants, but alterations in the liver-conditional knockout were far more widespread and intense (Figure 8). The characteristic pattern in both mutants was a sharp reduction of hepatic amino acid concentrations, which appear to be related to a relative depletion in the hepatic concentration of glutamate. Indeed, glutamate is the “pivot” of nearly all amino acid metabolism as it furnishes amino groups to ketoacids to support the concentrations of alanine, leucine, isoleucine, valine, and phenylalanine, as well as to glutamine via glutamine synthetase. In addition, glutamate rapidly enters the TCA cycle as α-ketoglutarate either via transamination or the glutamate dehydrogenase pathway. We propose that diminished glutamate may occur in *Pdss2* mutants due to their enhanced utilization of glutamate as a metabolic fuel in response to a relative diminution in their glucose consumption rate via glycolysis. It is recognized that in some tissues – particularly kidney and brain – glutamate can become an important metabolic substrate [23]. The significant reduction observed in alanine concentration (Figure 8, Panel C) is consistent with this formulation, since alanine carbon forms from pyruvate that is produced in glycolysis. Similarly, the sharp reduction in the glutamate∶aspartate ratio in mutant liver implies an increase of hepatic oxaloacetate, a TCA cycle intermediate which is the obligatory precursor both to aspartate carbon as well as to the TCA cycle citrate synthetase reaction. In other words, if glycolytic flux is relatively diminished in the *Pdss2* mutants, less pyruvate will be produced and flux through pyruvate dehydrogenase will be attenuated, thereby limiting availability of acetyl-CoA to the citrate synthase reaction, which would augment the hepatic pool of oxaloacaetate and impair TCA cycle flux. Diminished glutamate would also impede production of N-acetylglutamate, an obligatory effector of the carbamylphosphate synthetase reaction sequence in the mitochondrial segment of the urea cycle that leads from ammonia to citrulline, the total absence of which (Figure 8, Panel C) points to a compromise of ureagenesis in the liver of B6.*Alb/cre,Pdss2^loxP/loxP^* mice. It is of interest in this regard that the sole amino acid to be significantly increased is aspartic acid (Figure 8, Panel C), since a failure of citrulline synthesis would sharply curtail flux through cytosolic argininosuccinate synthetase, a step in the urea cycle that utilizes both citrulline and aspartate as precursors. The diminished glutamate∶aspartate ratio highlighted above also denotes compromised flux through the hepatic urea cycle, to which aspartate is a major nitrogen donor. Additional investigations are necessary and underway to verify the extent to which metabolic flux is impaired in *Pdss2* CoQ biosynthetic mutants.

We conclude that the *Pdss2^kd^* allele causes a milder phenotype in homozygous mice than that observed in the homologous human case [11]. What is especially puzzling about the *Pdss2^kd/kd^* mutant mouse is why an intermediate level of activity in oxidative phosphorylation should uniquely affect podocytes, a cell type not previously characterized by unusually high levels of respiratory activity. However, it is clear that CoQ concentrations and intermediary metabolism are significantly altered in the missense mutants. It is also interesting that the *Pdss2^kd/kd^* mouse is a mammalian model of mitophagy, a process recently described in yeast to involve the preferential degradation of impaired mitochondria before initiating cell death [24]. The occurrence of a subsequent intense inflammatory response suggests that autoantigens are exposed by autophagy to initiate either an innate or adaptive immune response. The inflammatory response is not the primary cause of the renal disease, but appears to be the mechanism used to collect and dispose of cellular debris. Availability of B6.*Pdss2^loxP/loxP^* mice will now permit future investigations into the specific sites of CoQ deficiency and tissue damage, particularly in the central nervous system and skeletal muscle, which lead to the heterogeneous but severe consequences of primary mitochondrial disease as observed in the human *PDSS2* patient.

## Materials and Methods

### Construction of Vectors

The targeting construct and *Pdss2^loxP/loxP^* construct were made as follows. The 11.8 kb linearized DNA containing the *Pdss2* exon 2 was from a bacterial artificial chromosome (BAC #RP23-256E1) with digestion of *Bam*H 1 and *Dra* III, and was subcloned into pBlueScript vector. The cassette containing loxP-neo-loxP was inserted into an *Aat* II site, and a loxP was introduced into an *Xho* I site. The *Pdss^loxP/loxP^* targeting construct is depicted in Figure 1 and contains approximately 7 kb of homology regions. This linear targeting construct was electroporated into R1 ES cells. Stably transfected cells were isolated after selection in G418 (350 ul/ml, Gibco), and 384 clones were screened for the desired homologous recombination event with PCR. Chimeric mice were generated by aggregation of ES cells with morulae of B6 mice, and the modified allele was passed through the germline by breeding chimeras to B6 mice. The genotypes of all offspring were analyzed by polymerase chain reaction using DNA from the ear of mice or the yolk sac of embryos. The k/oF and k/oR primers for determination of Exon 2 (PCR product of 440 bp) were 5′-GGAGGTTGAGTCCCTGTGTC-3′ and 5′- GCAGGAAATCAGTGGGACTC-3′ respectively. PCRs were carried out for 30 cycles (94°C for 20 s, 60°C for 30 s, 72°C for 1 min) in a buffer containing 1.5 mM MgCl_2_.

### Mice

Mice homozygous for the floxed gene (B6.*Pdss2^loxP/loxP^*) were crossed with partners that expressed *cre* under the control of a zona pelucida glycoprotein 3 promoter (C57BL/6-Tg(Zps-cre)93 Knw/J; *Zp3/cre*), obtained from The Jackson Laboratory (Bar Harbor, ME), which caused the floxed gene to be deleted in all tissues. Podocyte-specific knockouts (B6.*Podocin/cre,Pdss2^loxP/loxP^*), were obtained by crossing mice homozygous for the floxed gene with partners that expressed *cre* under the control of the *Podocin* promoter [25]. The mutation was targeted to the renal proximal tubular epithelial cells (as well as a subset of hepatocytes) by mating B6.*Pdss2^loxP/loxP^* mice with partners that express a *PEPCK/cre* transgene [26]; to hepatocytes by utilizing mice with an *albumin/cre* promoter (B6.Cg-Tg(Alb-cre)21 Mgn/J (*Alb/cre*)), obtained from The Jackson Lab; or cells of the myeloid lineage with mice that express *cre* under the control of the *lysozyme M* promoter (B6.129P2-*Lyzs^tm1(cre)Ifo^*/J; *LysM/cre* [27] obtained from The Jackson Laboratory. Mice with the *Pdss2^kd/kd^* missense mutation on the B6 background have been previously reported [7],[10]. All procedures were approved by the Institutional Animal Care and Use Committee of the University of Pennsylvania.

### Albumin Assay

A mouse albumin ELISA kit was obtained from Bethyl Laboratories Inc. Briefly, ELISA plates (Nunc, No: 442404) were coated overnight with goat anti-mouse albumin antibody (10 µl /ml) in bicarbonate buffer pH 9.6. Wells were washed with distilled water, blocked with PBS-1% BSA, washed with PBS-0.05% Tween 20, and 100 µl of serially diluted samples or standards added for 1 hour at room temperature. Plates were then washed using PBS-0.05% Tween 20, and incubated with 100 µl of HRP-conjugated goat anti-mouse albumin antibody at room temperature for 1 hour. After further washing with PBS-0.05% Tween 20, wells were incubated with 100 µl ABTS solution at room temperature for 20 min, then with 100 µl 2 M H_2_SO_4_ stopping solution and the plate was read with an ELISA reader at 405 nm.

### Histology

Kidneys from mutant and control mice were fixed in formalin, paraffin-embedded, and 4 µm sections through the longitudinal axis of each kidney were prepared and stained with hematoxylin and eosin. The sections were examined blindly and scored as follows: 0 = no tubular dilatation and no mononuclear cell infiltrates; 1 = small focal areas of cellular infiltration and tubular dilatation involving less than 10% of the cortex; 2 = involvement of up to 25% of the cortex; 3 = involvement of up to 50% of the cortex; 4 = extensive damage involving more than 75% of the cortex. Coronal sections of the brain were stained by H & E. To examine the retina, anesthetized mice were perfused through the heart with freshly prepared 2% PFA+2% glutaraldehyde in 0.2 M sodium cacadylate buffer (pH 7.4). Eyes were removed and eyecups were fixed in the same buffer for 4 hours at room temperature. Tissues were then dehydrated in a graded ethanol series, infiltrated, and embedded in EMbed812 (Electron Microscopy Sciences). Sections of 1–2 µ in thickness were cut and stained with toluidine blue. Liver sections were examined after H & E and Oil Red O staining. Skeletal muscle was stained by Gomori/trichrome stain, and with antibodies to ATPase, SDH, NADH, and acid phosphatase.

### Laser Capture Microdissection and PCR Amplification

Mouse kidney sections 6 µm in thickness were cut onto nuclease-free Membrane Slides for Laser microdissection (Molecular Machines and Industries, Lenor City VA). After hematoxylin staining, laser microdissection of glomeruli or collecting tubules was done using the SL µcut system, and samples were collected onto adhesive caps of 0.5 ml tubes as previously described [25]. Microdissected samples were incubated with 50 µl lysis buffer containing 10 mM Tris HCl (pH 8.3), 50 mM KCl, 2 mM MgCl_2_, 0.1 mg/ml gelatin, 0.45% NP40, 0.45% Tween 20, 6 µl/ml Proteinase K overnight at 55°C. 0.5 µl lysis buffer was used for each PCR reaction. Primers with the PCR product containing exon 2 of *Pdss2* were as follows: forward, 5′-AGCTGTGCACATGTGTGTGA-3′ and reverse, 5′-AAGCTTTTATGTGCCCGATG-3′. Primers with the PCR product containing exon 4 of *Pdss2* were: forward, 5′-TGCAGGAGGATTATCACAGC-3′ and reverse, 5′-TGCACATCAATTTTTCCCATT-3′.

### Electron Microscopy

As previously described [28], kidney tissue samples were fixed in 2.5% glutaraldehyde and 2.0% paraformaldehyde in 0.1 M sodium cacodylate buffer, pH 7.4, overnight at 4°C. After three cacodylate buffer washes, the samples were post-fixed with 2.0% osmium tetroxide in 0.1 M cacodylate buffer for one hour at 4°C. After two additional sodium cacodylate washes and a wash in dH_2_O, samples were stained with 2% aqueous uranyl acetate for 30 minutes at room temperature. Samples were rinsed again in H_2_O subsequent to dehydration in graded ethanols, and infiltration and embedding in Embed-812 (Electron Microscopy Sciences, Fort Washington, PA). All sections were examined in a JEOL 1010 electron microscope, and digital images were recorded with a Hamamatsu camera system.

### CoQ Measurements in Mouse Kidney and Liver Homogenates

Whole kidneys and livers were dissected from sacrificed mice and were stored at −80°C until homogenization. Individual kidneys or lobes of livers were placed in 5 mL of 1X PBS, pH 7.4 (137 mM NaCl, 8 mM Na_2_HPO_4_, 2.7 mM KCl, 1.5 mM KH_2_PO_4_) 4°C, and tissues were homogenized a total of 10 strokes with a tight-fitting Teflon pestle rotating at maximal speed with a Fisher Scientific Lab Stirrer LR400A. The homogenate was centrifuged at 1000×*g* for 5 minutes. The supernatants were removed to fresh vials, and protein concentrations were measured by the bicinchoninic acid assay (Pierce, Rockford, IL). Aliquots of each homogenate were transferred to 50 ml glass tubes and stored at −80°C until extracted. Lipid extractions were performed on CoQ_9_ standards, CoQ_10_ standards, and mouse tissue homogenates by adding the same amount of coenzyme CoQ_6_ internal standard along with 0.5 ml water, 9 ml methanol, and 6 ml petroleum ether to each. Mixtures were vortexed for 1 min, centrifuged at 910×*g*, and the top layer of petroleum ether was removed and saved in a separate vial. Fresh petroleum ether (6 ml) was added to each vial containing the aqueous phase and vortexed for 1 min. The vials were subjected to centrifugation as before and the second petroleum ether layer removed. The process was repeated once more, and the three pooled petroleum ether fractions were dried under nitrogen and resuspended in 200 µl methanol. The quinones were then separated and quantified by HPLC connected to an electrochemical detector as described [29], with the following exceptions: the precolumn electrode was the only electrode used and was set at +650 mV to oxidize all hydroquinones, and a Gilson 118 UV/Vis detector was utilized to detect quinones (275 nm) as they eluted from the column. The amount of CoQ_9_ and CoQ_10_ in the standards and samples was normalized to the amount of CoQ_6_ recovered in the individual lipid extracts.

### Integrated Respiratory Capacity Analysis of Freshly Isolated Liver Mitochondria

Mice were sacrificed by cervical dislocation without general anesthetic, since anesthetics may exacerbate mitochondrial dysfunction in primary mitochondrial disease [30]. Mouse liver mitochondria were isolated in a similar manner as described previously for rat liver mitochondria [31]. Briefly, livers were immediately dissected and collected in MSM buffer (220 mM mannitol, 70 mM sucrose and 5 mM MOPS, pH: 7.4). All subsequent mitochondrial isolation procedures were done on ice. Livers were washed twice with MSM, finely minced, and then homogenized in MSM-E (MSM containing 2 mM EDTA) with 2 slow strokes using a tight-fitting Teflon pestle in a glass Potter/Elvehjem tissue grinder (DuPont, Wilmington, Delaware) at 400 rpm using an IKA RW20 digital homogenizer (Cole-Palmer, Illinois). Following initial centrifugation at 300 g×10 minutes at 4°C, the supernatant was collected and centrifuged at 7000 g×10 minutes at 4°C (Eppendorf 5810R centrifuge, rotor F34-6-38, Hamburg, Germany). The pellet containing mitochondria was then washed twice in MSM with high speed centrifugation at 7000 g×10 minutes at 4°C. 2% protease inhibitor cocktail was added to the final washed pellet preparation (SIGMA, St. Louis, MO). Protein concentration was determined by Lowry assay [32]. Mitochondrial oxygen uptake by polarography using a clark-type electrode (Oxytherm, Hansatech Instruments, UK) was performed on freshly isolated mitochondria at 30°C, as previously described [30]. Substrates used were malate+glutamate, succinate, and TMPD+ascorbate to assess complex I-, II-, and IV- dependent integrated respiratory capacity, respectively. State 3 oxidative phosphorylation (OXPHOS) respiratory rates were measured in the presence of ADP to approximate maximal mitochondrial capacity. Mean state 3 rates with standard errors were calculated from at least duplicate tracings for each electron donor substrate for liver mitochondria isolated from 6 B6.*Pdss2^kd/kd^* missense mutants, 3 B6. *Alb/cre,Pdss2^loxP/loxP^* conditional knockout mutants, and a total of 9 concurrent age- and strain-matched controls (6×B6 and 3×B6.*Pdss2^loxP/loxP^*, respectively). As no statistically significant differences were detected in state 3 rates for any substrate between B6 and B6.*Pdss2^loxP/loxP^* controls, all control data (B6+B6.*Pdss2^loxP/loxP^*) were used for comparative analysis with each mutant strain. Comparison of means was done by one-way ANOVA assuming equal variance (SPSS v.12.0, Chicago, IL).

### Liver and Kidney Mitochondrial Enzyme Activity Analyses

Following polarographic analysis of isolated liver mitochondria, as described above, remaining mitochondrial aliquots were promptly frozen in −80°C and subsequently available for study on 4 B6.*Pdss2^kd/kd^* missense mutants, 2 B6.*Alb/cre,Pdss2^loxP/loxP^* conditional knockout mutants, and a total of 6 concurrent age- and strain-matched controls (4×B6 and 2×B6.*Pdss2^loxP/loxP^*, respectively). Duplicate frozen aliquots of each strain were studied, when available. Mitochondria from single whole kidney homogenates were isolated in a similar fashion as described above for liver mitochondria with the exception that the renal capsule was first manually removed by squeezing. As yields of mitochondria were insufficient to permit polarography with all substrates as described above, kidney mitochondria quality was assessed for 3 representative strains by polarography with malate+glutamate and succinate to demonstrate robust state 3 rates, respiratory control ratios > 3 and > 2, respectively, and ADP/O malate+glutamate > 3.5.

Enzyme assays for citrate synthase and the partial reactions of electron transport were performed at 37°C in a total reaction volume of 1 mL using standard methods [33]. Absorbance changes were continuously monitored using the dual-beam mode of an OLIS-converted DW2a spectrophotometer. Sensitivity to enzymatic inhibitors was used to confirm assay specificity. Complex I+III was measured at 550 nm minus 540 nm (extinction coefficient 19.0 mM^−1^ cm^−1^) as rotenone-sensitive NADH-cytochrome *c* oxidoreductase. Complex II+III was measured at 550 nm minus 540 nm 19.0 mM^−1^ cm^−1^) as antimycin A-sensitive succinate-cytochrome *c* oxidoreductase [7],[8]. Aliquots of mitochondria were preincubated with succinate for 10 minutes before assay of complex II+III. Complex III was measured at 550 nm minus 540 nm (19.0 mM^−1^ cm^−1^) using decylubiquinol as antimycin A-sensitive ubiquinol-cytochrome *c* oxidoreductase. Complex IV was measured at 550 nm minus 540 nm (19.0 mM^−1^ cm^−1^) as azide-sensitive ferrocytochrome c oxidase. Citrate synthase was measured at 412 nm minus 360 nm (13.6 mM^−1^ cm^−1^) using 5,5-dithio-bis(2-nitrobenzoic acid) to detect free sulfhydryl groups in coenzyme A. Individual enzyme activities are reported in nmol/min/mg protein. As no statistically significant differences were detected in activities for any substrate between B6 and B6.*Pdss2^loxP/loxP^* liver mitochondria controls, all control data (B6+B6.*Pdss2^loxP/loxP^*) were used for comparative analysis with each mutant strain. Comparison of means was done by paired t-test.

### *Pdss1* and *Pdss2* Expression Analysis

Standard precautions were followed to avoid contamination or degradation of RNA samples [34]. Total RNA was extracted from 30 to 90 mg aliquots of freshly isolated liver collected from each animal sacrificed for polarographic analysis (see above), and liver from an additional three animals each with genotypes B6.*Alb/cre,Pdss2^loxP/loxP^* B6.*Pdss2^loxP/loxP^* , B6, and B6.*Pdss2^loxP/loxP^* to verify *Pdss1* expression results. In addition, RNA was extracted from single whole kidneys of three B6.*Pdss2^kd/kd^* missense, three B6.*Podocin/cre,Pdss2^loxP/loxP^* mutant mice, three B6 controls, and three B6. *Pdss2^loxP/loxP^* controls using Trizol reagent (Invitrogen Corporation, Carlsbad, CA) and purified in RNeasy spin columns (Qiagen, Inc., Valencia, CA). Total RNA concentration and dissolution were determined spectrophotometrically at 230 nm, 260 nm, and 280 nm (NanoDrop ND-100 Spectrophotometer v3.1.2, NanoDrop Technologies, Inc., Wilmington, DE). 10 µg of total RNA was DNase-treated using a TURBO DNA-free kit (Ambion Inc., Austin, TX). 1.1 µg of DNase-treated RNA was reverse-transcribed in 20 µl reaction mixtures to generate cDNA using a High Capacity cDNA Reverse Transcription Kit (Applied Biosystems, Foster City, CA). 40 ng of cDNA was used per quantitative PCR (qPCR) reaction containing Taqman gene expression MGB probes with FAM dye-labeled assays for both endogenous (mouse *β-actin* Mm02619580_g1) and target (mouse *Pdss1* Mm00450958_m1 or *Pdss2* Mm01191894_m1) genes, as well as Taqman Universal PCR Master Mix (Roche, Branchburg, NJ), per Applied Biosystems standard protocol (Applied Biosystems, Foster City, CA). Real-time analysis was performed on an SDS-7500 qPCR machine (Applied Biosystems, Foster City, CA). Sequence Detection Software 1.2.3 version was used for relative quantitation gene expression analysis (Applied Biosystems, Foster City, CA). Relative gene expression is reported as the mean and range of individual mutant strains each compared with pooled age- and strain-matched controls.

### Biochemical Pathway Expression Profiling Microarray Analysis

Aliquots of total RNA were prepared from the same 3 B6.*Alb/cre,Pdss2^loxP/loxP^* mutants and 3 B6.*Pdss2^loxP/loxP^* controls subjected to mitochondrial studies as described above. The *C. elegans* Genome Array was utilized for expression microarray analysis (Affymetrix, Inc, Santa Clara, CA). Affymetrix probe-level data was normalized and summarized in dChip using PM-only model to obtain a single log2-transformed intensity value per probe set per array (hyyp://biosun1.harvard.edu/complab/dchip). The processed data was sent to gene set enrichment analysis (GSEA) for cluster analysis while genes were ranked by the built-in signal-to-noise function (GSEA v. 2.0, Broad Institute, Cambridge, MA). Clusters were designed to represent *M. musculus* genesets relevant to human metabolic pathways. Genesets were curated from biochemical pathway data publicly available from Kyoto Encyclopedia of Genes and Genomes (KEGG) (www.genome.jp/kegg). While 102 KEGG pathways were identified, only 95 genesets contained 5 or more transcripts to meet criteria for inclusion in the analysis. *C. elegans* microarray analysis was performed as previously reported well-characterized mitochondrial complex I, II, and III missense and RNA-interference generated hypomorphic mutants [21]. All microarray expression data is available in a publicly available MIAME-compliant database (GEO superSeries GSE9967 for *C. elegans* arrays and GEO accession GSE10904 for *M. musculus* arrays).

### Quantitative Amino Acid Profiling in Liver

Wedges of liver (∼400 mg) were immediately frozen in −80°C and homogenized in 4 ml aliquots of 4% perchloric acid that contained ε-aminocaproic acid (100 µM) as internal standard. After centrifugation to remove protein, supernatants were adjusted to pH 6–7 with KOH, then centrifuged again to remove precipitated KClO_4_. Amino acids were measured in the resulting supernatant by high performance liquid chromatography (HPLC) using precolumn o-phthalaldehyde derivatization with fluorescent detection (Varian, Palo Alto, CA) [35]. The protein pellet was dissolved in 1N NaOH and results were normalized to protein as determined by Lowry assay [32]. Means were compared with a Student's *t*-test.
